# Editorial: Insights into gender, sex, and sexualities: 2022

**DOI:** 10.3389/fsoc.2024.1378891

**Published:** 2024-03-08

**Authors:** Kath Woodward

**Affiliations:** Department of Sociology, The Open University, Milton Keynes, United Kingdom

**Keywords:** gender, sexualities, identification, sex, culture, insights

The 13 different articles in this Research Topic demonstrate the enduring relevance and importance of gender, sex, and sexualities in making sense of social relations and social change. Gender is a concept that can be used to explain sociocultural inequalities and the relationships between different axes of power, which generate inequalities in social systems, and as an empirical mode of classification that places people, often within a binary logic that has dominated Western culture, into one of two categories, i.e., as female or male. However, this is changing fast, due not least to the challenge to the binaries of sex and gender and of male and female. Change is recognized and addressed in the range of articles in this Research Topic. Historically, sex and gender have been theorized as separate and each has been given a different weighting, with biological sex being seen as shaping sociocultural gender. This view is challenged here; for example, in the context of non-binary and trans identities in child development by Salinas-Quiroz and Sweder, who explore what they term the authenticity of non-binary gender identification from an early age. They posit an argument that weighs up the impact of different influences, the availability of role models, and representations on children's perception of themselves as outside the gender binary. They consider how children who do not identify with existing feminine and masculine traits can be themselves agents in self-identification as non-binary. Their approach, like those of other authors in this Research Topic, demonstrates the nuances and complexities of understanding gender, sex, and sexualities outside the nature-nurture dichotomy.

The study of sexualities, including sexual orientation, increasingly manifests blurred boundaries between gender and sexuality, reflected in those on LGBTQI+ identities here. Comeau et al. extend the categories to include 2S, which brings in specific local cultural practices among indigenous people in Canada, in this case, the idea that a person can be two-spirited with feminine and masculine spirits. They demonstrate the specificities of the incidence and experience of health problems among gender minority groups, illuminating the interaction between social factors and health.

The articles in this Research Topic engage with transformations as well as acknowledging continuities in thinking about gender, sex, and sexualities and living with gender.

A diverse range is important to this Research Topic as its major concern is change in thinking about and understanding gender in an area of fast-moving change. The discussion ranges from conversations between feminism and the legacy of machismo in Peru in the context of representations of music and popular musicians [the cumbia singers Marisol and Rosado, who perform versions of the Latin American music with its percussion-heavy beat and fusion of rock and folk (Gutiérrez-Gómez and Munaris-Parco)], to mental healthcare for young and adolescent LGBTQ+ individuals who experienced particular difficulties accessing care during the COVID-19 pandemic in the Indian sub-continent (Sanjeevkumar Gaur et al.). Some research is based upon global empirical statistical evidence; for example, of adolescent pregnancies (Fute et al.), which demonstrates the interrelationships between individuals and wider society and between personal experience and politics and policies, which could alleviate problems in both arenas. These articles illustrate the scope and fluidity of gender, sex, and sexualities, especially the practice, policies, and experiences of them across a wide range of social terrains.

Gender plays out in different ways but remains ubiquitous in that gender matters in shaping social and sexual relations and impacting upon experience, which makes gender a shared focus of the Research Topic, which covers a very wide range of empirical sites. The Research Topic includes an empirical and theoretical mix of subjects and specific locations and intersections with different social forces. Many of the contributions focus on health. Health care can involve prejudice against people who identify outside the gender binary as well as those occupying different socioeconomic classes and subject to other forms of exclusion. de Dios-Aguado et al. argue that the endurance of syphilis, a sexually transmitted disease that can be cured, can be attributed to discrimination against marginalized people who are not always able to access care and treatment for a variety of reasons. Sedighi et al.'s study explores reasons why relationship counseling is largely not offered to people with multiple sclerosis, and their partners report a lack of sexual satisfaction in the relationship. Other articles suggest more positive policy solutions; for example, in the case of a systematic review of digital interventions for young LGBTQ+ people with mental health problems (Liu et al.). Although these young people experience discrimination, the authors argue that digital intervention offers a more positive policy intervention than approaches deployed hitherto. At a more personal individual level, the role of sexting as an adaptive coping mechanism in couple relationships improved the wellbeing of Italian women during the second wave of the COVID-19 pandemic (Bonfanti et al.).

The issue of the rights of marginalized gender and sexual identities is also a concern of articles in this Research Topic. Rodrigues et al. argue that human rights are not available to trans people in Brazil and Portugal, rendering people “non-human.” Rights and the denial of particular gender identities and sexual orientations can be affective in their impact. Feelings and emotions are included in the mix in analyses in the Research Topic. As Di et al. argue in the case of reactions to acute events, which they claim produce greater levels of stress in women than men. Gender still imprints a sexist logic on the organization of data, which has policy implications. Politics and personal feelings are connected in this field of research. Stigma produces a gay glass ceiling thus limiting promotion possibilities for gay people in Salvati et al.'s study of employment and promotion in Mexico. Additionally, Wicker and Cunningham's study suggests that affect matters in sport, which is dominated by gender binaries and gender stereotypes; however, club membership can mitigate the constraints of stereotypes and generate more democratic less stereotypical participation by mitigating the more oppressive aspects of stereotyping.

The studies in this Research Topic engage with the uneven pace of change in different parts of the world and with the diversity of lived experience and policies at a time when new identities and ways of thinking about gender, sex, and sexualities are on the agenda and are part of everyday life.

## Author contributions

KW: Writing—review & editing, Writing—original draft.

